# Supplementary material for: Examining the association between fetal HLA-C, maternal KIR haplotypes and birth weight
Source: PLoS Genet. 2026 Apr 20;22(4):e1012102. doi: 10.1371/journal.pgen.1012102 (PMC13095029; doi:10.1371/journal.pgen.1012102)
Supplement: S1 Text — (PDF) [file pgen.1012102.s002.pdf]

Supplementary Information

Estimating the proportion of variance explained by the main effect and interaction reported in Hiby et al. (2014)

Hiby et al. report that the frequency of *KIR2DS1* in the English population is approximately p = 0.2. It follows that approximately 0.2^2 + 2*0.2*0.8 = 0.36 of mothers should be positive for *KIR2DS1*. Hiby et al. report that presence of *KIR2DS1* is associated with an 84g increase in birth weight. Thus, the locus explains approximately 2*p*(1-p)*β^2 = 0.36*(1 - 0.36)*84^2 = 1625.7024 g^2 variance. Assuming the standard deviation of birth weight is roughly 500g, the phenotypic variance is around 250000g^2. This translates to the locus explaining 0.65% of the variance. Most complex trait loci explain <0.1% of the variance in quantitative traits and this has certainly been the case for birth weight, however, a locus that explains 0.65% of the variance is not outside the realms of possibility.

In order to estimate the proportion of variance explained by the interaction reported in Hiby et al., we need to work out the expected proportion of pregnancies where the offspring possesses more *HLA-C2* alleles than their mother. Assume that the frequency of *HLA-C2* is p = 0.3. This is equivalent to saying that 30% of offspring carry an *HLA-C2* allele from their father. For the 30% of offspring who carry this allele transmitted from their father, their mothers can be homozygous for the other allele with probability (1 - p)^2 = 0.7^2 = 0.49, or heterozygous and transmit the other allele to their offspring with probability p*(1 – p) = 0.7*0.3 = 0.21. Thus, the expected proportion of pregnancies where the offspring possesses more *HLA-C2* alleles than their mother is 0.3*(0.49 + 0.21) = 0.21.

As explained above, we expect 36% of mothers to be positive for *KIR2DS1*. Assuming independence, the proportion of offspring who should carry an *HLA-C2* allele from their father and have a mother positive for *KIR2DS1* = 0.21*0.36 = 0.0756. Hiby et al. report that the interaction confers a 245g increase in birth weight. Thus, the locus explains 0.0756*(1 – 0.0756)*245^2 = 4194.8 g^2 variance. This translates to the interaction explaining 1.68% of the phenotypic variance.

Estimating Power to Detect a Main Effect of KIR2DS1 and an Interaction Between Maternal KIR2DS1 and Fetal HLA-C2 In Our Study Via Simulation

We simulated genotypes at the *KIR2DS1* locus assuming an allele frequency of p = 0.2 for 10,602 mothers. We simulated a phenotype (offspring birth weight) where maternal *KIR2DS1* explained 0.65% of the phenotypic variance in offspring birth weight according to a dominant model. We simulated so that birth weight had unit variance. We then ran a test of association regressing offspring birth weight on the number of copies of the risk allele in mothers. We calculated power across 1000 simulations.

We examined the effect of imputation error on the power to detect an association. We parameterized accuracy as the probability of correctly imputing the correct *KIR2DS1* genotype. Genotypes that “failed” imputation were randomly changed to one of the other two genotype categories. We estimated power (two tailed α = 0.05) across 1000 simulations where birth weight was regressed on the number of copies of the *KIR2DS1*

allele. **Table A** shows that we would expect to maintain high power to detect an effect of *KIR2DS1* even with substantial decreases in imputation accuracy (i.e. far larger than what would be the case in our study). Intuitively, this is because our study is an order of magnitude larger than the original Hiby et al. publication.

Table A. Estimated power to detect an association between maternal *KIR2DS1* and offspring birth weight with varying levels of imputation accuracy using simulation.

KIR Imputation Accuracy	Power To Detect Association
100%	100%
95%	100%
90%	100%
85%	99.9%
80%	99.5%
75%	95.9%
70%	89.5%

To simulate the interaction, we assumed that the frequency of the birth weight increasing biallelic *HLA-C2* allele was p = 0.3. We simulated transmission of the variant in 10,602 parent-offspring trios and independently simulated the biallelic *KIR2DS1* locus as before in mothers. We simulated offspring birth weight to have unit variance and assumed that the interaction between maternal *KIR2DS1* and offspring *HLA-C2* explained 1.68% of the phenotypic variance. Specifically, offspring birth weight increased only when mothers were positive for *KIR2DS1* and their offspring carried more *HLA-C2* alleles than their mothers. For each replicate, we regressed offspring birth weight on three terms, the number of copies of *KIR2DS1* in mothers, a binary term indicating whether the offspring carried more *HLA-C2* alleles than their mothers, and a term representing an interaction between the two terms. We calculated the significance of the interaction term. Power was defined as the number of statistical tests of the interaction term reaching significance across 1000 simulations (two tailed $\alpha = 0.05$). We examined the effect of imputation error on the power to detect association as before.

Again, power to detect the interaction was extremely high and did not decrease measurably even with the addition of substantial imputation error in maternal *KIR2DS1* (power > .99 with only 80% accuracy).

R code detailing the simulations is appended below.

R Code

```
###MAIN EFFECT###
set.seed(123)

### PARAMETERS
n <- 10602
p <- 0.2
nsim <- 1000
alpha <- 0.05
var_explained <- 0.0065

### Genotype probabilities under HWE
geno_probs <- c((1-p)^2, 2*p*(1-p), p^2)

### Dominant indicator variance
q <- 1 - (1-p)^2
varD <- q * (1 - q)

### Effect size giving 0.65% variance explained
beta <- sqrt(var_explained / varD)

### Residual SD so phenotype variance = 1
sigma <- sqrt(1 - var_explained)


##################################################
# FUNCTION: run simulation with optional error
##################################################

simulate_power <- function(accuracy = 1){

  pvals <- numeric(nsim)

  for(i in 1:nsim){

    ### Simulate genotype (0,1,2)
    geno <- sample(0:2, n, replace=TRUE, prob=geno_probs)

    ### Dominant model for true phenotype
    D <- ifelse(geno > 0, 1, 0)

    ### Simulate phenotype
    y <- beta * D + rnorm(n,0,sigma)

    ### Introduce genotyping error
    geno_obs <- geno

    wrong <- runif(n) > accuracy

    if(any(wrong)){
      geno_obs[wrong] <- sapply(geno_obs[wrong], function(g){
        sample(setdiff(0:2, g), 1)
      })
```

```
    }

    ### Association test (additive model)
    fit <- lm(y ~ geno_obs)

    pvals[i] <- summary(fit)$coef[2,4]
  }

  power <- mean(pvals < alpha)

  return(power)
}


#################################################
# POWER WITHOUT GENOTYPING ERROR
#################################################

power_true <- simulate_power(accuracy = 1)

power_true


#################################################
# POWER WITH DIFFERENT GENOTYPING ACCURACY
#################################################

accuracy_values <- seq(0.7,1,0.05)

power_results <- sapply(accuracy_values, simulate_power)

results <- data.frame(
  accuracy = accuracy_values,
  power = power_results
)

results
```

###R CODE FOR INTERACTION###

```
set.seed(123)

#############################
# PARAMETERS
#############################

n <- 10602
p_kir <- 0.2
p_hla <- 0.3
var_explained <- 0.0168
nsim <- 1000
alpha <- 0.05
```

```
##############################
# GENOTYPE SIMULATION
##############################

simulate_genotype <- function(n, p){
  probs <- c((1-p)^2, 2*p*(1-p), p^2)
  sample(0:2, n, replace=TRUE, prob=probs)
}


##############################
# HLA TRANSMISSION
##############################

simulate_trios <- function(n, p){

  mother <- simulate_genotype(n, p)
  father <- simulate_genotype(n, p)

  transmit <- function(g){
    sapply(g, function(x){
      if(x==0) return(0)
      if(x==2) return(1)
      rbinom(1,1,0.5)
    })
  }

  off_m <- transmit(mother)
  off_f <- transmit(father)

  offspring <- off_m + off_f

  list(mother = mother,
       offspring = offspring)
}


##############################
# PHENOTYPE SIMULATION
##############################

simulate_phenotype <- function(kir_mother, hla_mother, hla_offspring){

  kir_dom <- as.numeric(kir_mother > 0)

  hla_diff <- as.numeric(hla_offspring > hla_mother)

  interaction <- kir_dom * hla_diff

  vg <- var(interaction)

  beta <- sqrt(var_explained / vg)

  genetic <- beta * interaction
```

```
  residual_sd <- sqrt(1 - var_explained)

  y <- genetic + rnorm(length(genetic), 0, residual_sd)

  return(list(y=y,
              kir_dom=kir_dom,
              hla_diff=hla_diff))
}


##############################
# GENOTYPING ERROR FUNCTION
##############################

introduce_error <- function(g, accuracy){

  g_obs <- g

  incorrect <- runif(length(g)) > accuracy

  if(any(incorrect)){
    g_obs[incorrect] <- sapply(g_obs[incorrect], function(x){
      sample(setdiff(c(0,1,2), x), 1)
    })
  }

  g_obs
}


##############################
# ASSOCIATION TEST
##############################

test_interaction <- function(y, kir_add, hla_diff){

  fit <- lm(y ~ kir_add + hla_diff + kir_add:hla_diff)

  summary(fit)$coefficients["kir_add:hla_diff",4]
}


##############################
# POWER WITHOUT ERROR
##############################

pvals <- numeric(nsim)

for(i in 1:nsim){

  kir_mother <- simulate_genotype(n, p_kir)

  trio <- simulate_trios(n, p_hla)
```

```
  hla_mother <- trio$mother
  hla_offspring <- trio$offspring

  sim <- simulate_phenotype(kir_mother, hla_mother, hla_offspring)

  pvals[i] <- test_interaction(sim$y,
                               kir_mother,
                               sim$hla_diff)
}

power_no_error <- mean(pvals < alpha)



#############################
# POWER WITH GENOTYPE ERROR
#############################

accuracy <- 0.80

pvals_error <- numeric(nsim)

for(i in 1:nsim){

  kir_true <- simulate_genotype(n, p_kir)

  trio <- simulate_trios(n, p_hla)

  hla_mother <- trio$mother
  hla_offspring <- trio$offspring

  sim <- simulate_phenotype(kir_true, hla_mother, hla_offspring)

  kir_obs <- introduce_error(kir_true, accuracy)

  pvals_error[i] <- test_interaction(sim$y,
                                     kir_obs,
                                     sim$hla_diff)
}

power_with_error <- mean(pvals_error < alpha)


#############################
# RESULTS
#############################

cat("Power without genotyping error:", power_no_error, "\n")
cat("Power with KIR2DS1 genotyping accuracy =", accuracy, ":",
power_with_error, "\n")
```
