# Supplementary material for: Examining the association between fetal HLA-C, maternal KIR haplotypes and birth weight
Source: PLoS Genet. 2026 Apr 20;22(4):e1012102. doi: 10.1371/journal.pgen.1012102 (PMC13095029; doi:10.1371/journal.pgen.1012102)
Supplement: S2 Fig — Effects represent the estimated change in offspring birth weight (g) for each additional maternal KIR2DS1 allele (A) in the presence of fetal HLA-C2, (B) in the presence of more HLA-C2 alleles in the fetus relative to the mother, and (C) when fetal HLA-C2 is paternal in origin; total n = 7,437. UKB, UK Biobank; EFSOCH, Exeter Family Study of Childhood Health; HAPO, Hyperglycemia and Adverse Pregnancy Outcomes study; ALSPAC, Avon Longitudinal Study of Parents and Children; BiB, Born in Bradford. (PDF) [file pgen.1012102.s004.pdf]

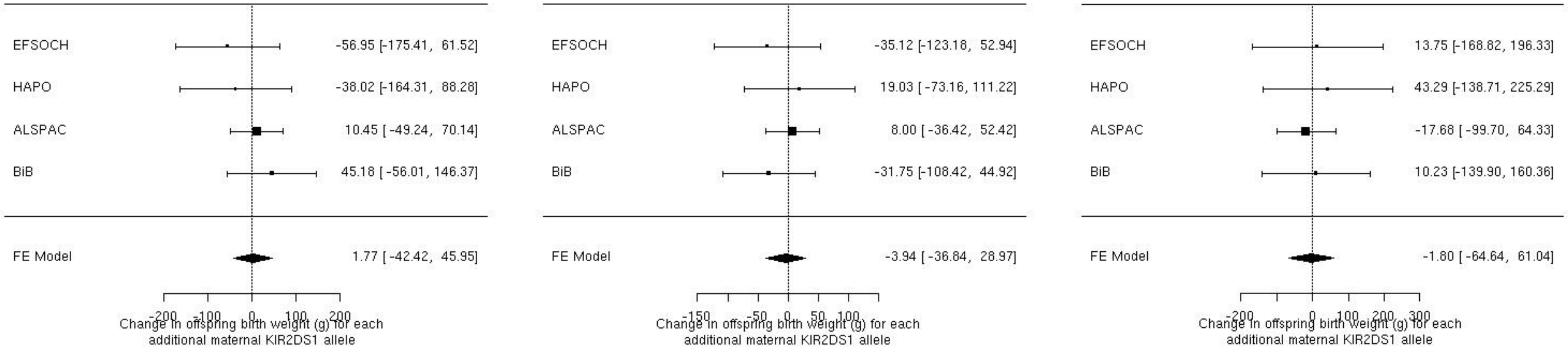

A **B** **C**

S2 Fig. Meta-analysis omitting the UK Biobank (UKB) cohort (the only sample with birth weight not corrected for gestational age) to assess impact on the association between maternal *KIR2DS1 alleles*, fetal *HLA-C*, and birth weight. Effects represent the estimated change in offspring birth weight (g) for each additional maternal *KIR2DS1* allele **(A)** in the presence of fetal *HLA-C2*, **(B)** in the presence of more *HLA-C2* alleles in the fetus relative to the mother, and **(C)** when fetal *HLA-C2* is paternal in origin; total n = 7,437. UKB, UK Biobank; EFSOCH, Exeter Family Study of Childhood Health; HAPO, Hyperglycemia and Adverse Pregnancy Outcomes study; ALSPAC, Avon Longitudinal Study of Parents and Children; BiB, Born in Bradford.
