# Supplementary material for: Examining the association between fetal HLA-C, maternal KIR haplotypes and birth weight
Source: PLoS Genet. 2026 Apr 20;22(4):e1012102. doi: 10.1371/journal.pgen.1012102 (PMC13095029; doi:10.1371/journal.pgen.1012102)
Supplement: S7 Fig — (A) The estimated imputation accuracy achieved at each KIR locus. (B) The distribution of the posterior probabilities of the most likely alleles for each KIR locus where the distribution of the input dataset is shown in blue against the reference panel distribution shown in red. (PDF) [file pgen.1012102.s009.pdf]

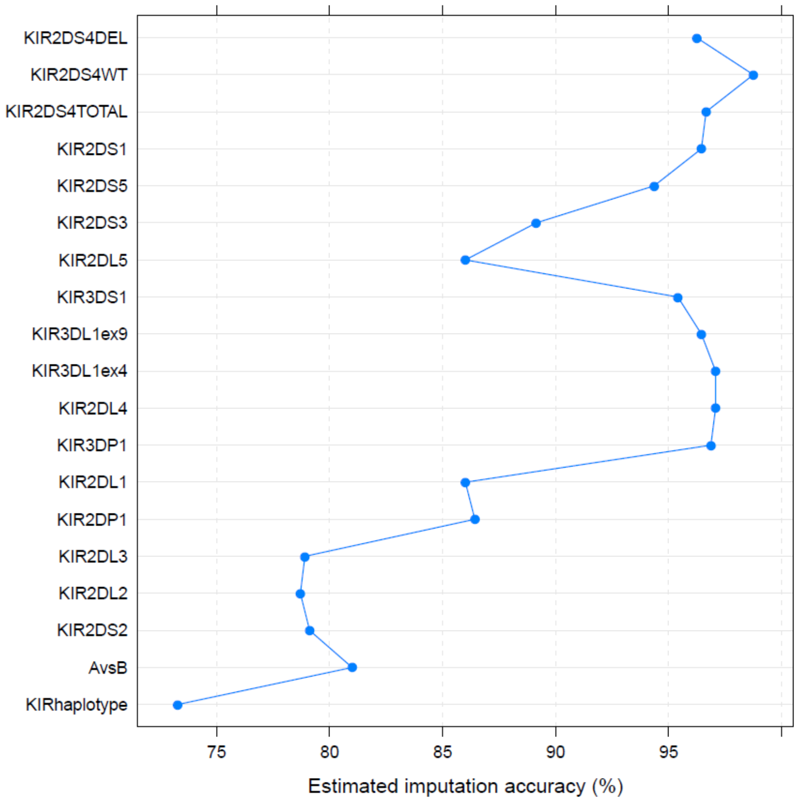


**A**

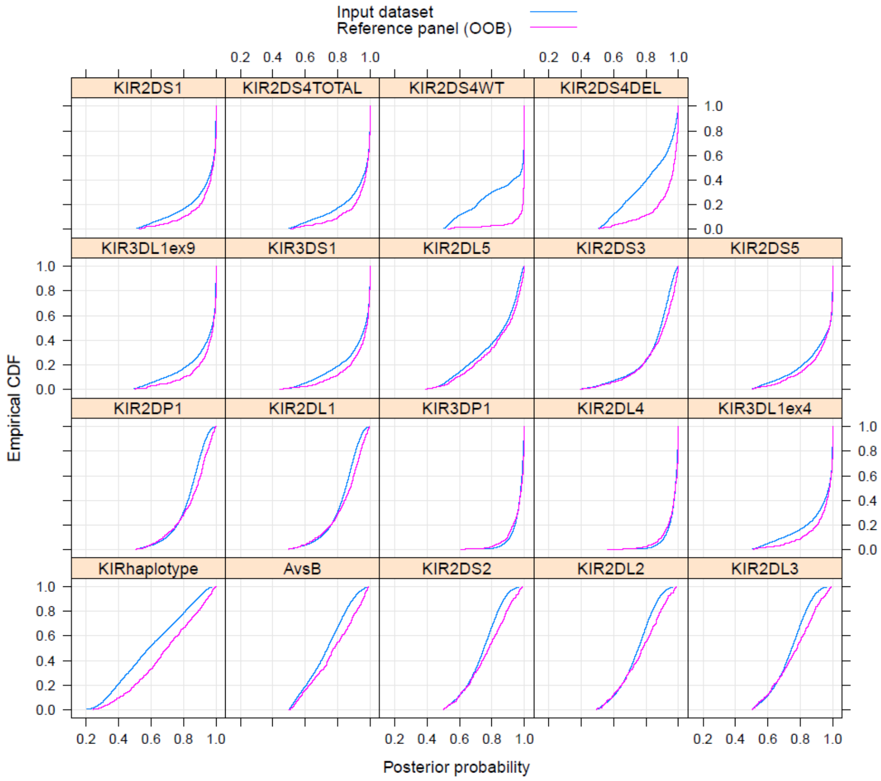


**B**

**S7 Fig. KIR imputation plots for the Born in Bradford (BiB) Core Exome mother-offspring pairs (n=5,709 at imputation stage). (A)** The estimated imputation accuracy achieved at each KIR locus. **(B)** The distribution of the posterior probabilities of the most likely alleles for each KIR locus where the distribution of the input dataset is shown in blue against the reference panel distribution shown in red.
