# Supplementary material for: Examining the association between fetal HLA-C, maternal KIR haplotypes and birth weight
Source: PLoS Genet. 2026 Apr 20;22(4):e1012102. doi: 10.1371/journal.pgen.1012102 (PMC13095029; doi:10.1371/journal.pgen.1012102)
Supplement: Attachment — Submitted filename: ResponseToReviewers_PLOSGen_2025_11_23.pdf [file pgen.1012102.s012.pdf]

Responses to Reviewers' Comments

Submission ID: PGENETICS-D-25-00434
Corresponding author: Caitlin S. Decina
Title: Examining the association between fetal HLA-C, maternal KIR haplotypes and birth weight

We thank all reviewers for their thoughtful comments and suggestions. We have responded to these below and feel that the manuscript is strengthened as a result. Throughout this document the reviewers' comments have been renumbered and the text bolded, followed by our responses. Text added to the manuscript is stated below its corresponding response with page and line numbers referring to the resubmitted manuscript document. A marked-up version of the revised manuscript showing tracked changes has also been uploaded as a related file.

<u>Reviewer 1</u>

This manuscript has used several cohorts to look at the association of maternal KIR and fetal HLA-C variants with birthweight. The previous studies looking at this question have only had few samples. They have now used birthweight data from ~10,000 mother-offspring pairs from 5 different cohorts. The KIR and HLA polymorphisms have been imputed. There are several issues that need addressing:

1. The cohorts are all slightly different. The gestational age is known, and the birth weight has been corrected for this in 4 cohorts, but not in UKB where the birthweight is also self-reported. Gestational age is clearly strongly correlated with birth weight. This means this cohort should be left out in their analysis to see if the results are altered.

We acknowledge the reviewer's concern for the relevance of gestational age in our analysis; this information was unfortunately unavailable to correct for in the UK Biobank (UKB) cohort. In the figures below, we present our three models of fetal *HLA-C2* interaction with dosage of maternal *KIR B* haplotype (**Figure R1**) and dosage of maternal *KIR2DS1* (**Figure R2**) when omitting the UKB sample from our meta-analysis as a sensitivity analysis. When comparing **Figures R1** and **R2** and **Figures 1** and **2**, respectively, in the manuscript, we observe some fluctuation in the overall meta-analyzed effect estimates, however, all estimates ultimately remain null with large confidence intervals. This is not unexpected given the overall low amount of observable heterogeneity in effect sizes between the cohorts that is seen in **Figures 1** and **2** in the manuscript, and is particularly visible in the plots below, where the effects in all cohorts overlap the null with wide confidence intervals.

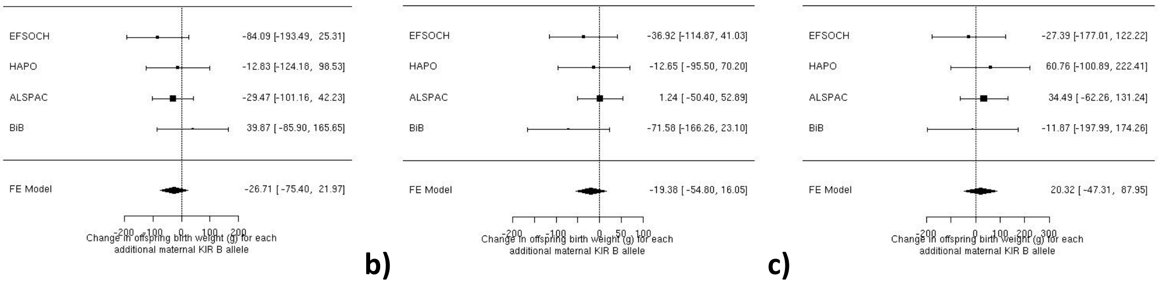

a) **b)** **c)**

Figure R1. Meta-analysis of the estimated change in offspring birth weight (g) for each additional maternal *KIR B* allele **a)** in the presence of fetal *HLA-C2*, **b)** in the presence of more *HLA-C2* alleles in the fetus relative to the mother, and **c)** when fetal *HLA-C2* is paternal in origin; total n = 7,437.

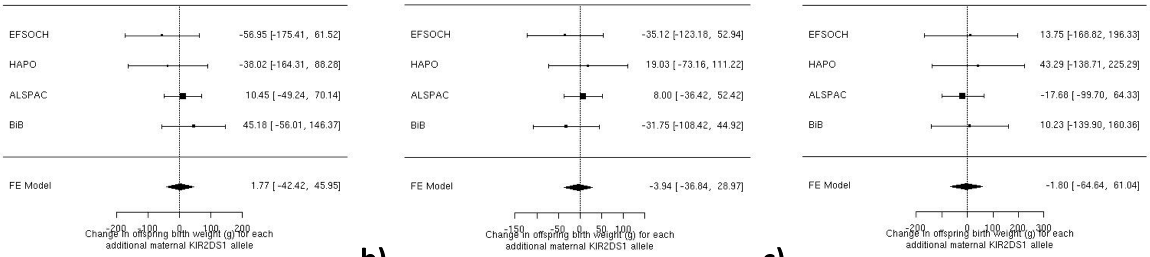

a) **b)** **c)**

Figure R2. Meta-analysis of the estimated change in offspring birth weight (g) for each additional maternal *KIR2DS1* allele **a)** in the presence of fetal *HLA-C2*, **b)** in the presence of more *HLA-C2* alleles in the fetus relative to the mother, and **c)** when fetal *HLA-C2* is paternal in origin; total n = 7,437.

Thus, including UKB in our analysis, despite lack of correction for gestational age in this sample, does not alter our findings or conclusions. As such, we feel that it is appropriate for the UKB dataset to remain in the meta-analysis as reported, however, for reassurance for readers we have now added these plots as supplementary figures (now S1 and S2) and included in the manuscript that upon performing this sensitivity analysis our results are unchanged:

Results, page 17, lines 369-370: "When performing a sensitivity analysis removing the UKB cohort, as the only sample with birth weight not adjusted for gestational age, the results were unchanged (see **S1 Fig and S2 Fig**)."

2. How were these pairs corrected for other known factors that influence birth weight – for example: BMI, fetal sex, first pregnancy. It would be helpful to have a flow chart of how all the exclusions were made.

Our analyses were corrected for fetal sex, gestational age (except UKB), genotyping batch, principal components, and year of birth (UKB only due to the wide range of birth years compared to the other cohorts). Though maternal BMI and parity are known to influence offspring birth weight, neither should affect genotype and confound this relationship and is therefore not necessary to control for. We adjusted for sex to reduce variability in the birth

weight outcome, not because we expect it to be a confounding factor. Our interest was to replicate the findings of Hiby et al. 2014 in which their study also did not adjust for parity or heritable traits such as BMI, and only corrected for fetal sex, gestational age, and cohort. We extended their covariate adjustments mainly through the use of principal components to guard against any effects of population stratification.

Below we have created a flow chart outlining the general procedure for how the datasets were prepared with a focus on the exclusion steps performed at the final phenotypic variable preparation and filtering stage prior to association modelling.

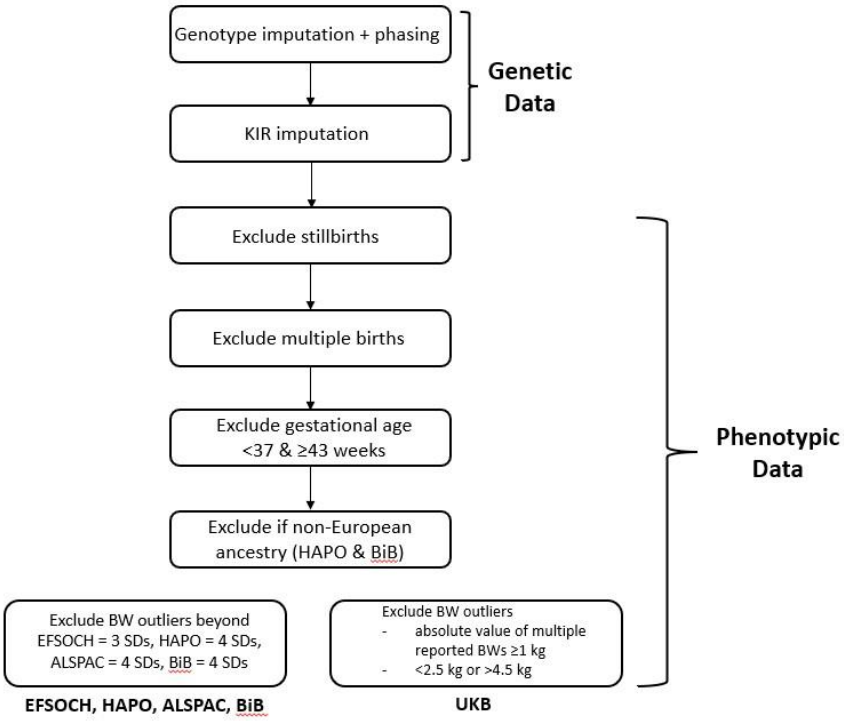

3. It seems that birth weights at the extremes of the normal distribution have been excluded (<2.5Kg and >4.5Kg). As birth weight is under stabilizing selection this presents a significant problem as the effects would be expected at these extremes. Related to this is that the previous studies on KIR and HLA-C in pregnancy have mainly looked at pregnancies presenting with clinical problems. These have mainly been preeclampsia and intra-uterine growth restriction – those at one end of the birth weight range. Thus, the question posed in this study is rather different than the question addressed in the study they are supposedly disproving. It is perfectly possible that KIR and HLA-C variants have little influence in the middle of the birth weight spectrum. They are studying birthweights that are not at the extremes - but all previous studies have focussed on the extremes. To reiterate, this study uses continuous variable EXCLUDING extremes with the assumption that the effect is linear, which is very likely not to be the case.

The reviewer is correct that birth weights at the extremes of the normal distribution have been excluded in our study. In the case of UKB, birth weights <2.5kg and >4.5kgs were removed to reduce bias from possible reporting errors and, to the best of our ability, exclude preterm births since information on gestational age was not available (lines 301-303 in the original manuscript). It is also true that much of the studies that established the relationship between maternal *KIR* and fetal *HLA-C* genotypes used pregnancy samples characterized by pathologies or complications such as pre-eclampsia[1], fetal growth restriction[2], and recurrent miscarriage[2,3]. However, it is not true that all previous studies have focussed on birth weight extremes. The specific study we sought to replicate, Hiby et al. 2014[4], investigated the effect of maternal *KIR* and fetal *HLA-C* combinations on increased birth weight using a sample of all pregnancies >5th centile, which included high birth weights but predominantly the range of normal birth weight (while excluding pregnancies with fetal growth restriction and pre-eclampsia). Where we may differ from their study is perhaps not including birth weight as high as was included in their sample, though they do not indicate the actual measured range of included birth weights in grams to compare.

With respect to the assumption of linearity, Hiby et al. 2014 produced their main novel finding analyzing birth weight as a continuous variable using linear regression, as we have done in our analysis. Their previous work and accompanying analyses within their 2014 paper indicate a likely linear relationship between certain *KIR*, *HLA-C*, and birth weight i.e. a trend of high maternal *KIR AA*, low activating *KIR2DS1* frequency in pregnancies associated with poor placentation (birth weight ≤5th centile and pre-eclampsia) and low *KIR AA*, high *KIR2DS1* frequency in high birth weight pregnancies, with mothers of average birth weight babies having intermediate *KIR* frequencies. As such and given their finding of a large increase in birth weight of ~250g for the combination of maternal *KIR2DS1* and fetal *C2* of paternal origin, we would expect to see some effect of increased birth weight even within the spectrum of normal birth weight if this relationship were true, as we mention in our discussion. As the reviewer has suggested, we do however leave room for the possibility that any influence of *KIR* and *HLA-C* variants may only be observable in complicated pregnancies and birth weight extremes, which we acknowledge in our discussion on lines 417-420 of the original manuscript. The purpose of our study and research question was to test the association of these variants within the birth weight spectrum, largely in the normal range, as was done by Hiby et al. 2014.

As a sensitivity analysis, we have tested the inclusion of previously excluded birth weight outliers in our UKB sample to see if our associations are strengthened in the direction of an increase in birth weight among the tested combinations. These results are presented in the table below with comparison to our reported effect estimates plotted in **Fig 1** and **Fig 2** in the manuscript. We see here that the addition of these outliers does not significantly change the observed relationship and would not suggest an effect only in outlier individuals which the reviewer suggests.

		UKB BW excluding extremes (range = 2.5Kg – 4.5kg)		UKB BW including extremes (range = 0.57 – 6.35kg)	
**Model***	**Genetic Effects Modelled**	**Effect Estimate†**	***P*-value**	**Effect Estimate***	***P*-value**
(1)	Presence of fetal C2<br>Maternal *KIR B*<br>Interaction	-25.29	0.28	-16.02	0.58
(2)	Presence of fetal *C2*<br>Maternal *KIR2DS1*<br>Interaction	-11.62	0.65	-1.52	0.96
(3)	Amount of fetal *C2*<br>Maternal *KIR B*<br>Interaction	12.57	0.48	11.54	0.60
(4)	Amount of fetal C2<br>Maternal *KIR2DS1*<br>Interaction	27.10	0.16	17.56	0.46
(5)	Paternal fetal *C2*<br>Maternal *KIR B*<br>Interaction	-11.32	0.73	2.83	0.94
(6)	Paternal fetal *C2*<br>Maternal *KIR2DS1*<br>Interaction	-12.73	0.72	4.78	0.91

*N=3,434 in models (1) and (2); N=3,415 in models (3) and (4); N=3,430 in models (5) and (6)
†The estimated change in offspring birth weight (g) for each additional maternal allele

4. The work of Sinsheimer PMID: 26567478 PMID: 28214848 is not cited and must be considered as GWAS for quantitative traits can miss genetic associations. These models need to be included as they might show model specification and power. There might be too many variables in their models to properly test for interaction - mixing cohorts, parity, gestational age.

The QMFG model developed and tested in the work of Clark et al. 2016 and 2017[5,6] (Sinsheimer group) serves as an association testing method for maternal-fetal genotype (MFG) interactions that is particularly robust in cases of offspring-only datasets of quantitative traits typically produced by GWAS studies, where use of such data may lead to missed or misattributed associations when MFG effects exist. In our study, we avoid the pitfalls of such data in the first instance by using genotyped data from cohort studies with mother-child pairs, rather than summary data from GWAS' that may only capture offspring genotypes.

Clark et al. 2017 extend the QMFG test to multiple loci to investigate the same relationship between maternal *KIR2DS1*, more fetal *HLA-C2* than the mother, and birth weight as in Hiby et al. 2014 using the same sample of white European individuals. They expand this replication by specifically testing an interaction between the loci and if there are additional independent effects of the loci. The main QMFG model they describe which tests this, Model 1 in their paper, is the model found to best fit this data and assess these relationships, which models the genetic effects of maternal *KIR2DS1*, more *HLA-C2* in the fetus, and an interaction between them. This is essentially Model 4 in our study presented in **Table 2** and **Fig 2b** of the manuscript, testing the same genetic effects with both loci included in the model as well as

an interaction term. With respect to covariate adjustment, we adjusted our tested models for offspring sex and gestational age (except UKB) as they did, with the added benefit of adjustment for mother and child principal components in our models to guard against effects of population stratification. Additionally, in terms of statistical model type, the QMFG model they built is a linear mixed model – the same as those used in our study. Clark et al. 2017 indeed state that “When the data consist of only mother-offspring pairs, general statistical software packages that include linear mixed model options can be used”, as we have done for our study. Therefore, we feel confident that our analyses already consider the points of concern their modelling methods seek to address, and our methods closely align with those they used to test for interaction in the sample from Hiby et al. 2014. Following this, it is important to highlight that although Clark et al. 2017 were able to replicate an association between maternal *KIR2DS1*, greater *HLA-C2* in the fetus, and increased birth weight as in the Hiby et al. 2014 study, they used the same sample of ~1300 mother-child pairs while our study increased sample size by approximately a factor of 10. Therefore, regardless of model similarities or differences, the impact of sample size on results must be considered and their study may be just as likely to have found a false positive due to the small sample. We have added the following to the discussion to the acknowledge the Sinsheimer group’s work and its relation to ours:

Discussion, pages 20-21, lines 419-443: “Previous work has been done to determine modelling methods that most accurately capture maternal-fetal genotype (MFG) interactions, such as that by Clark et al. 2016 and 2017, which developed the extended quantitative-MFG (QMFG) test, a linear mixed model that can apply familial multi-locus genotype data. Their model was designed to address the issues of low power or incorrect conclusions obtained when using standard models that only consider offspring genotypes. In the Clark et al. 2017 study, they used this model to the investigate the same relationship between maternal *KIR2DS1*, more fetal *HLA-C2* than the mother, and birth weight as in Hiby et al. 2014, using the same sample of white European individuals (n≈1,300 mother-child pairs). They expanded this replication by specifically testing an interaction between the loci and if there are additional independent effects of each locus. They found that the model that best fit this data and assessed these relationships, models the genetic effects of maternal *KIR2DS1*, more *HLA-C2* in the fetus, and an interaction between them (denoted as Model 1 in their study). With this model, they found a significant *KIR–HLA-C* interaction effect on birth weight that explained ∼1% of the phenotypic variation in birth weight. The model they used is essentially Model 4 in our study presented in **Table 2** and **Fig 2b**: a linear mixed model testing the same genetic effects with both loci included as well as an interaction term. With respect to covariate adjustment, we adjusted our tested models for offspring sex and gestational age (except UKB) as they did, with the added benefit of adjustment for mother and child principal components in our models to guard against effects of population stratification. We have thus used a method previously determined to be the most appropriate to assess the maternal-fetal genetic relationships at these loci but were unable to replicate an effect in our analysis. It is important to reiterate that although Clark et al. 2017 were able to replicate an association between maternal *KIR2DS1*, greater *HLA-C2* in the fetus, and increased birth weight as in the Hiby et al. 2014 study, they used the same small sample of mother-child pairs while our study

increased sample size by approximately a factor of 10. Therefore, regardless of model similarities or differences, the impact of sample size on results must be considered."

5. Key to this study is the typing at these extraordinarily polymorphic loci. KIR*IMP is still not considered robust by many labs. Only one method has so far been published to impute KIR reflecting the difficulties in doing this. It is stated that imputation accuracy is >95% but how is this determined? To confirm the findings, it is essential to type at least 25% of samples by routine KIR typing to confirm their accuracy of imputation. What is the percentage of correct calls when this has been done? Do their imputed KIR reflect published findings in the populations tested in the cohorts?

We thank the reviewers for their comments and questions. For clarity, the error rate reported for KIR imputations is the out-of-bag error (OOB) obtained from the random forest training at the heart of KIR*IMP, using exactly the SNPs we used for imputation. We have clarified this in the text (lines 235-237; see overall amendment below). We note four main points, which we address in turn: (i) HLA imputation using HLA*IMP has been shown to be extremely accurate using both cross-validation and independent data. It is completely accepted for studies of this nature; (ii) Out-of-bag (OOB) error (obtained from the random forest model at the heart of HLA*IMP:03 and KIR*IMP) concords extremely well with error rates obtained from cross-validation, so is a suitable proxy for the error rate; (iii) KIR*IMP uses the same model as HLA*IMP:03, and here we use it in a completely analogous manner, meaning we have confidence that the OOB error rate is a strong reflection of the true underlying error rate. We have further tested this using cross-validation; and (iv) even allowing for some inaccuracy, we have shown that the reduction in power in a large cohort study will not dramatically affect the findings.

(i) The method for HLA imputation, HLA*IMP:02, is the gold-standard for typing HLA from SNP genotype data and has been used to type millions of samples. We have shown in well-powered analyses that it is accurate in all practical settings, see Motyer et al. 2016[7]. HLA*IMP:02 was used to type all the samples in the UK Biobank (UKB) for the main data release[8]. The accuracy of the UKB typing has been assessed independently on multiple occasions (see for example Krishna et al. 2024[9], which independently assesses the quality of the UK Biobank HLA imputations using exome sequence data from 43,000 UKB individuals), finding strong concordance between the genotype and sequence-based types. The UKB HLA types are accepted in the broader genetics community. Further validation seems excessive.

(ii) OOB error concords extremely well with cross-validation error in exactly this setting (shown for KIR*IMP in Vukcevic et al. 2015[10]). Experience with HLA*IMP:03, comparing cross-validation to OOB error supports this finding.

(iii) KIR*IMP uses the same model as HLA*IMP:03. HLA*IMP:03 is the most accurate method for typing HLA alleles and has been extensively tested on large data sets (see Motyer et al. 2016[7]), showing high accuracy across ethnicities. This gives us confidence that KIR*IMP will be accurate in the current setting. There are no

suitable independent KIR-typed data sets to test this in any other way. We note, we are only testing the simplest cases for KIR*IMP, and these have been shown by cross-validation to be extremely accurate. The error rates quoted in the paper use OOB error when building the model for the exact SNPs in our cohorts. (We note, for intellectual property reasons, we were unable to use HLA*IMP:03 to type HLA alleles in our cohorts, but benchmarking of the methods used in our setting demonstrate they give suitable high accuracy. See the methods own papers and Motyer et al. 2016[7]).

(iv) The paper by Motyer et al. 2016[7] assesses the effect of using imputations, with various levels of inaccuracy, on the power to detect an association. It shows that for large sample sizes (as we have here), the effect on power of some inaccuracy of HLA or KIR imputations for common alleles is small. Considering HLA, our major focus is on C1 versus C2 alleles, and these are accurately imputed. For KIR, of focus is KIR2DS1, which is the most accurately typed locus by KIR*IMP, with cross-validation estimates giving 99% accuracy.

For clarity, we have included some additional details in the Methods section to better introduce the KIR*IMP methodology, including the OOB error rates that determine KIR imputation accuracy. For better flow of information to readers, we also moved the description of the KIR*IMP software to the beginning of the "Imputation of *KIR* types" subsection, now followed by the description of our data preparation and imputation procedure. These changes are as follows:

Methods, pages 10-11, lines 230-239: "Imputation of KIR types was performed using the KIR*IMP software. KIR*IMP provides estimates of gene copy number, KIR A or B haplotype, as well as gene-content haplotypes for each individual using a reference panel of 301 SNPs constructed from a U.K. cohort of European ancestry. Using the SNP intersection between the input dataset and the reference panel, i.e. only the SNPs shared between the input dataset and the reference panel, the imputation software fits a random-forest model to the reference panel using only the intersecting SNPs. The software outputs the imputed KIR types with the most likely allele and its associated posterior probability at each locus along with estimates of the average imputation accuracy for each KIR locus. The accuracy estimates are the out-of-bag (OOB) accuracy calculated during the model-fitting process, which are shown to mimic cross-validation error rates and thus provide reliable estimates of KIR imputation performance – see Vukcevic et al. 2015."

Regarding the comment "Key to this study is the typing at these extraordinarily polymorphic loci. KIR*IMP is still not considered robust by many labs" we note that we are not attempting to type the highly polymorphic alleles of KIR, but merely the presence/absence or dosage of the gene KIR2DS1, which the KIR*IMP paper (Vukcevic et al. 2015[10]) shows is the easiest to type (i.e. is typed most accurately; 99%). Similarly, all studies show HLA*C is the easiest to type and we are typing the aggregated C1/C2 alleles.

It is both practically and financially unfeasible for us to type 25% of the sample by routine KIR typing.

(i) Performing routine KIR typing is not possible in the first instance as we do not have access to DNA samples for any of the cohorts. Access to biological samples, if possible to acquire at all for the cohorts we have included, requires extensive upfront organization and approval – not something we are able to undertake as a validation procedure at the post-analysis stage.

(ii) The time and financial cost of even attempting to do this is prohibitive and was beyond the scope of our objectives to use widely available data sources and scalable methods.

(iii) This is not necessary – the methods we apply have been used routinely across the field and have been validated in suitable settings (see above).

6. Only KIR2DS1 has been imputed but what about other activating KIR, especially KIR2DS4?

All KIR types were included in our imputation and obtained within the standard output of the KIR*IMP server. KIR2DS1 was, however, the sole locus of interest as this is the activating receptor that Hiby et al. 2014 demonstrated as having an association with increased offspring birth weight when in combination with fetal HLA-C2 of paternal origin – the relationship we sought to investigate in a larger sample.

Though KIR2DS4 has been found to have activating action on trophoblast cells through cytokine signalling activity, particularly when expressed together with KIR2DS1[11], KIR2DS4 binds a limited number of HLA-C1 and -C2 allotypes[12]. KIR2DS4 also has two different forms: KIR2DS4wt, the full-length form which binds some HLA-C, or KIR2DS4del, a truncated disabled form due to a 22-bp frameshift deletion[13]. The truncated form is present on ~75% of A haplotypes, with the functioning full-length form present in a relative minority of people[13,14]. The KIR2DS4 locus is therefore not excluded from contribution to pregnancy outcomes and is perhaps worth consideration for future study but appears to be of lesser importance than KIR2DS1 and was not relevant to our specific investigation, which was limited to what was tested previously.

7. Several groups have shown how uterine NK cells can alter invasive trophoblast behaviour that is influenced by KIR activation, but this work is not cited.

We acknowledge that when mentioning the interaction of HLA and KIR molecules' ability to modify trophoblast activity in the uterine wall, specifically in our introduction on **page 4, lines 81-83**: "HLA-C binding with KIRs regulates uNK function and trophoblast invasion into the uterine wall by producing either positive or negative signals via cytokine secretion depending on whether the KIR possesses activating or inhibitory function", we have only cited one source as demonstrating this relationship. We have now added an additional three references for this introductory material to support the existence of this relationship between HLA, KIR, and

trophoblast behaviour, and acknowledge the work of others who have provided functional evidence.

Reviewer #2

The authors report an attempt to replicate the previously described effect of the maternal KIR haplotype on offspring birth weight, particularly in cases where the offspring inherited the paternal HLA-C2 haplotype. They have substantially increased the sample size by meta-analysing five independent cohorts of mother–offspring pairs, improving upon the original study by Hiby et al. (2014). While the introduction and methods are well structured, the results are somewhat disappointing, as no significant associations were observed. Nevertheless, it is valuable to publish well-conducted negative findings, particularly when they challenge previously reported associations, and such efforts should be more strongly encouraged in the field.
Overall, the authors did a good job of describing their experiments and discussing why and how their results differed from previously reported associations.

8. My main concern is the heterogeneity and potential limitations in how KIR and HLA imputation was performed across cohorts. The entire study relies on imputed KIR and HLA data, which the authors report as accurate (Table 4). However, it is unclear how imputation accuracy was evaluated. From my understanding, the best way to assess accuracy is to downsample WGS data to SNP array sites, impute back, and compare to the WGS calls. I assume WGS data were only available for UKBB, if at all — so what benchmark was used to report accuracy?

We address the methodology used to determine accuracy/error rates as well as how this compares in terms of cross-validation and with well-established imputation methods in our response to comment #4 by Reviewer #1 above. Though our imputation methods varied in terms of software used, this was to prioritize maximum SNP overlap between the reference panel and input data in the case of KIR (important for obtaining the best possible accuracy estimates) and largely to accommodate differences in data management between cohorts (UKB using their own centralized methods) and data use limitations (e.g. prohibited data sharing to external servers for ALSPAC and BiB) in the case of HLA. As stated in the response to comment #4 above, despite some heterogeneity of methods, those we have used for imputation should be reliably robust.

Regarding using WGS data to validate our KIR imputation accuracy metrics, there is currently no suitably accurate method for using WGS to type KIR copy number. PING (software used for genotyping KIR loci) does not run properly on standard WGS data. Moreover, we are limited by our access to this data (available only in UKB) and thus our ability to perform this type of validation across cohorts. As we have noted in our response to comment #4 above, sequence data has been used to check the HLA*IMP:02 imputations and has shown these to be very

accurate. As we know HLA*IMP:03 is even more accurate, and KIR*IMP uses the same model, we do not think further testing in UKB is necessary.

9. In the Study population section, it is described that each independent study was quality-controlled and imputed. For some studies (e.g., BiB), a post-imputation quality filter is specified (variants with imputation info score < 0.3 were removed). Was this also applied to the other cohorts?

For BiB, this threshold was used as the genotype imputation was performed prior to this study at a different centre and is only being reported here. We did not implement this threshold ourselves, only applied this imputed genotype data to the KIR*IMP server. For the other studies, we did not apply a standard threshold to all or specifically assess quality score for all SNPs prior to KIR imputation.

10. It is also not entirely clear what was used as input for the KIR and HLA imputation. In the KIR*IMP manuscript, phased SNP array data are used as input. From reading your methods, it seems that you instead used imputed data as input. If so, I have a few follow-up questions:

a) Why not use only genotyped variants as input? Was there insufficient overlap with the reference panel?

We chose to use imputed genotype data to maximize overlap with the KIR*IMP reference panel and provide the most information possible to inform *KIR* type estimation. We did not explicitly test and compare the results when using only raw genotyped variants vs. imputed genotypes as we inferred that more available information would produce better *KIR* imputation outcomes. However, we found in certain cohorts that the imputed genotypes produced better or worse *KIR* type estimations depending on the reference panel used for genotype imputation. This was the case for the EFSOCH and HAPO cohorts for which we initially had available genotype data imputed against the Haplotype Reference Consortium (HRC) reference panel, which produced suboptimal *KIR* imputation results (only 239 and 238 SNPs, respectively, overlapped the 301-SNP KIR reference panel). When we instead imputed the genotype data for these cohorts ourselves against the TOPMed reference panel, *KIR* accuracy estimation was improved along with the posterior probabilities (256 and 258 overlapping SNPs, respectively), which we ultimately brought forward for our analysis. Results such as these, shown below in **Figures R3** for EFSOCH and **R4** for HAPO, highlight the need for good variant overlap with the KIR reference panel. Thus, using only raw genotyped variants as input would lack the information added by genotype imputation with an optimal reference panel and produce poorer KIR results similar to those seen when using the HRC imputed genotypes in these examples.

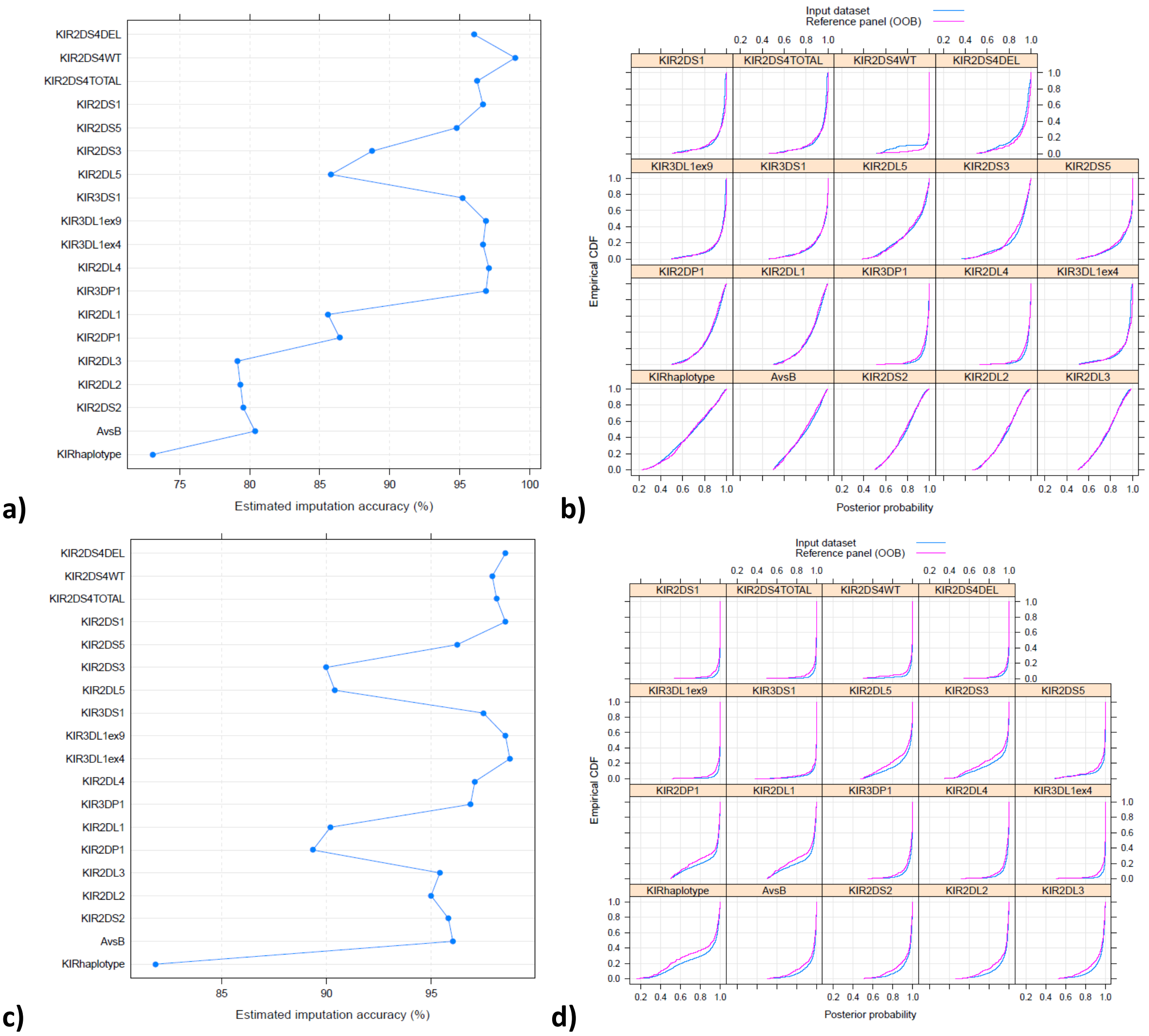

Figure R3. *KIR* imputation accuracy and posterior probability estimates for EFSOCH mother-child pairs using genotypes imputed to the HRC reference panel (**a** and **b**) compared to the TOPMed reference panel (**c** and **d**).

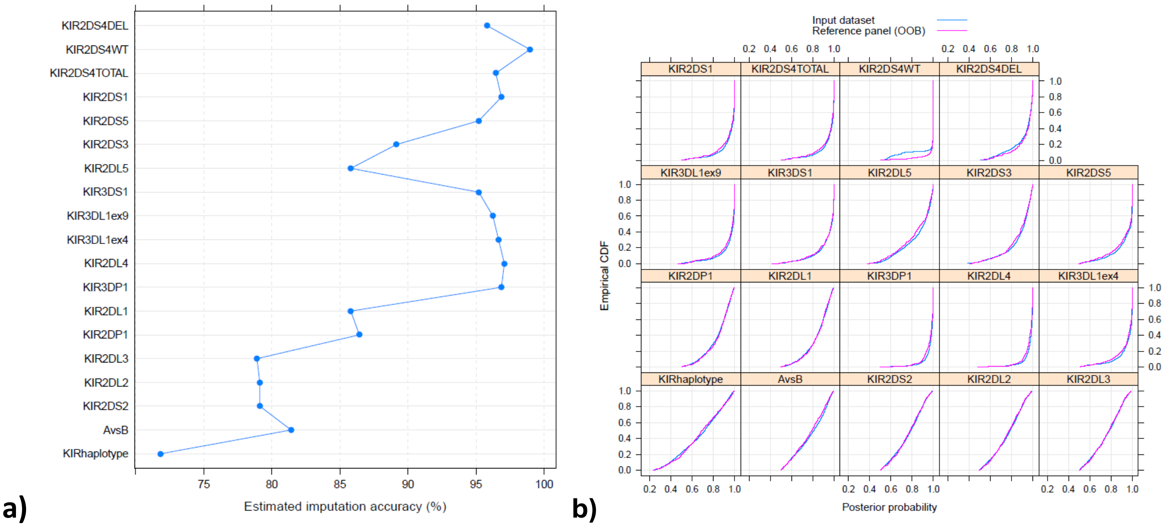

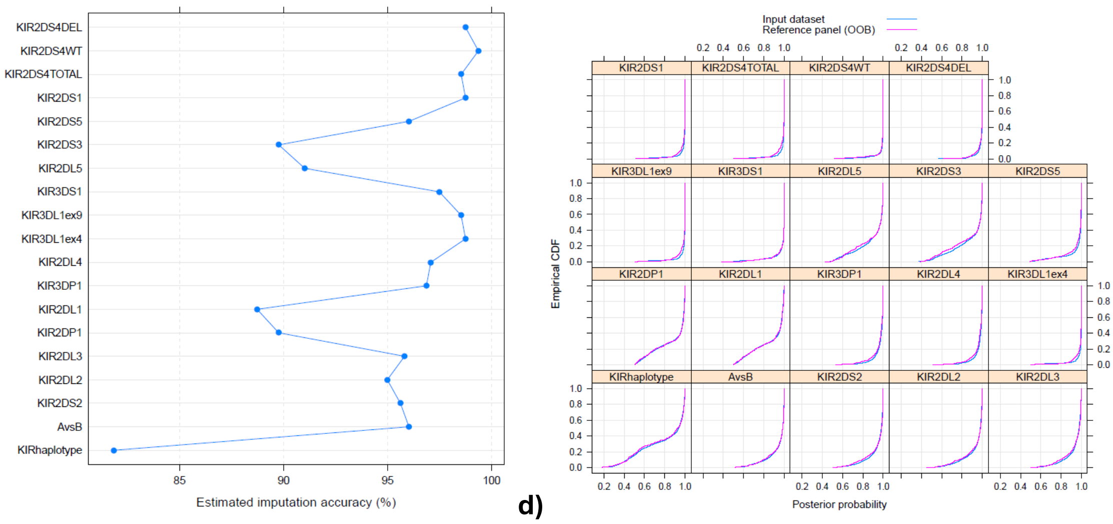

c) d)

Figure R4. *KIR* imputation accuracy and posterior probability estimates for HAPO mother-child pairs using genotypes imputed to the HRC reference panel (**a** and **b**) compared to the TOPMed reference panel (**c** and **d**).

b) Was the imputed data used as input already phased? Some imputation tools, such as IMPUTE5, output phased posterior probabilities — is this also the case when using the Michigan Imputation Server? From my understanding, KIR*IMP requires phased input.

Yes, the reviewer is correct that the KIR*IMP imputation software requires phased input. As such, the genotype imputed data was indeed phased prior to KIR imputation. Both the Michigan and TOPMed imputation servers offer the option to return phased genotypes using Eagle v2.4 and we selected this option as part of the genotype imputation process. This is explicitly stated on **lines 141** for the UKB sample, **line 183** for HAPO, and **line 226** for BiB. Though the same procedure is followed for the EFSOCH and ALSPAC samples we did not specifically mention that phasing was also performed for these samples and so we have now added that to the methods text for consistency on **lines 159-160** (EFSOCH) and **201** (ALSPAC).

c) An imputation quality filter of 0.3 seems rather permissive. Many GWAS use a threshold of 0.8. In your case, since the imputed data were used as input for KIR imputation, I believe it would be even more important to apply stringent quality control, for example using only variants with info score > 0.99, which should still retain enough markers for KIR imputation.

We agree with the reviewer that good SNP quality is important for informing good quality downstream imputation. Beyond poor SNP quality, KIR imputation quality also declines, however, with poor levels of overlap with the KIR imputation reference panel. Our priority, therefore, was to retain as many SNPs as possible and maximize the amount of overlap between our data and the SNPs that make up the KIR reference panel (301 SNPs constructed

from a U.K. cohort of European ancestry). In the table below, we show the number of SNPs ultimately kept in each cohort and used to inform KIR imputation, as well as how many SNPs within each set have an info score below 0.99 and below 0.8. We can see that if we applied a stringent quality control threshold of info score >0.99, we would lose a substantial number of SNPs in each cohort and very likely not retain enough markers for good quality KIR imputation. What is reassuring, however, is that for most of the cohorts, there are few or no SNPs below a quality threshold of 0.8. ALSPAC and BiB have the greatest number of SNPs that fall below this as we were limited by the coverage of this region on the genotyping chip. As such, it would not have been possible to include these cohorts in analyses if applying a threshold as high as 0.99.

	**Cohort**					
**KIR*IMP Reference Panel SNP Overlap**	**UKB**	**EFSOCH**	**HAPO**	**ALSPAC**	**BiB CE**	**BiB GSA**
Total No. SNPs Kept	259	256	258	235	229	230
No. SNPs Info Score <0.99	70	181	106	124	206	158
No. SNPs Info Score <0.8	6	0	0	25	87	31

Abbreviations: UKB, UK Biobank; EFSOCH, Exeter Family Study of Childhood Health; HAPO, Hyperglycemia and Adverse Pregnancy Outcomes study; ALSPAC, Avon Longitudinal Study of Parents and Children; BiB, Born in Bradford; CE, Core Exome; GSA, Global Screening Array.

Minor comments:

11. Line 288: How was the parental origin of the fetal HLA-C2 determined, particularly when both mother and offspring were heterozygous C1/C2?

Parental origin of fetal *HLA-C2*, specifically in the case of inferred inheritance from the father, was determined in the same manner as described in Hiby et al. 2014 where they defined this group based on mother-child pairs where the mother was homozygous for *C1* (*mC1C1*) and the fetus was heterozygous (*fC1C2*) and therefore fetal *C2* must be paternal in origin. As this is the only combination where paternal inheritance could be inferred unambiguously, mother-child pairs where both mother and offspring were heterozygous were not included in these models. We have added this clarification in the methods as follows:

Methods, page 13, lines 299-301: "... previously investigated by Hiby et al. 2014. For inferred inheritance of fetal *HLA-C2* from the father, mother-child pairs where both mother and offspring were heterozygous (*mC1C2/fC1C2*) were not included in the analysis as paternal origin could not be determined unambiguously."

12. Since mothers are genotyped, could you also explore the effect of the transmitted versus untransmitted maternal haplotypes?

Yes, this could be an avenue of investigation, however this was beyond the scope of what we sought to examine with the present study. Our main aim was to perform a replication of previous findings, testing the associations originally performed by Hiby et al. 2014 to determine whether an effect of maternal *KIR* and fetal *HLA-C2* on offspring birth weight could be seen when using larger birth cohort samples available today, along with reliable statistical methods for estimating KIR types without the need for resource- or cost-intensive procedures. Looking further into effects of maternal haplotype transmission could be an interesting aspect to explore in a future study.

In summary, I think this is a well-written and valuable study that addresses an important question with a much larger sample size than the original work. Although the lack of association is disappointing, this careful attempt to replicate the previous findings clearly shows how important replication is in genetic association studies, especially since the original study did not include any independent replication. Publishing well-conducted negative results, like in this case, is crucial and should be encouraged more in the field.

We thank the reviewer for this positive final comment. We agree that performing independent replication of genetic association studies is important and that the publication of negative results or null findings is informative to the research landscape. This is the key contribution of this study.

Reviewer #3

The manuscript by Decina et al examines the previous reported association between fetal human leukocyte antigen-C (HLA-C) and maternal killer immunoglobulin-like receptor (KIR) haplotypes on birth weight. This is important, as lower and higher birth weights have implications on health outcomes for both the mother and newborn. Previous associations with lower sample sizes suggested a strong association. The current study reexamines this association with a much larger cohort and finds no association. This is an important finding that will be of broad interest to human geneticists and physicians alike. The manuscript is well written, and the methods used are nicely described. The authors acknowledge that the cohort is skewed toward individuals of European descent and further studies are needed to thoroughly examine other cohorts.

We thank the reviewer for taking the time to evaluate our study. We appreciate their positive feedback regarding the quality of the manuscript in addition to the contribution of the findings and level of interest it will hold for the scientific community.

References

1. Hiby SE, Walker JJ, O'Shaughnessy KM, Redman CWG, Carrington M, Trowsdale J, et al. Combinations of maternal KIR and fetal HLA-C genes influence the risk of preeclampsia and reproductive success. Journal of Experimental Medicine. 2004;200:957–65. https://doi.org/10.1084/jem.20041214

2. Hiby SE, Apps R, Sharkey AM, Farrell LE, Gardner L, Mulder A, et al. Maternal activating KIRs protect against human reproductive failure mediated by fetal HLA-C2. Journal of Clinical Investigation. 2010;120:4102–10. https://doi.org/10.1172/JCI43998

3. Hiby SE, Regan L, Lo W, Farrell L, Carrington M, Moffett A. Association of maternal killer-cell immunoglobulin-like receptors and parental HLA-C genotypes with recurrent miscarriage. Hum Reprod. 2008;23:972–6. https://doi.org/10.1093/humrep/den011

4. Hiby SE, Apps R, Chazara O, Farrell LE, Magnus P, Trogstad L, et al. Maternal KIR in Combination with Paternal HLA-C2 Regulate Human Birth Weight. The Journal of Immunology. 2014;192:5069–73. https://doi.org/10.4049/jimmunol.1400577

5. Clark MM, Blangero J, Dyer TD, Sobel EM, Sinsheimer JS. The Quantitative-MFG Test: A Linear Mixed Effect Model to Detect Maternal-Offspring Gene Interactions. Ann Hum Genet. 2016;80:63–80. https://doi.org/10.1111/ahg.12137

6. Clark MM, Chazara O, Sobel EM, Gjessing HK, Magnus P, Moffett A, et al. Human Birth Weight and Reproductive Immunology: Testing for Interactions between Maternal and Offspring KIR and HLA-C Genes. Hum Hered. 2017;81:181–93. https://doi.org/10.1159/000456033

7. Motyer A, Vukcevic D, Dilthey A, Donnelly P, McVean G, Leslie S. Practical Use of Methods for Imputation of HLA Alleles from SNP Genotype Data. bioRxiv. 2016;091009.

8. Bycroft C, Freeman C, Petkova D, Band G, Elliott LT, Sharp K, et al. The UK Biobank resource with deep phenotyping and genomic data. Nature. 2018;562:203–9. https://doi.org/10.1038/s41586-018-0579-z

9. Krishna C, Tervi A, Saffern M, Wilson EA, Yoo SK, Mars N, et al. An immunogenetic basis for lung cancer risk. Science (1979). 2024;383:1–18. https://doi.org/10.1126/science.adi3808

10. Vukcevic D, Traherne JA, Næss S, Ellinghaus E, Kamatani Y, Dilthey A, et al. Imputation of KIR Types from SNP Variation Data. Am J Hum Genet [Internet]. The Authors; 2015;97:593–607. https://doi.org/10.1016/j.ajhg.2015.09.005

11. Kennedy PR, Chazara O, Gardner L, Ivarsson MA, Farrell LE, Xiong S, et al. Activating KIR2DS4 Is Expressed by Uterine NK Cells and Contributes to Successful Pregnancy. The Journal of Immunology. 2016;197:4292–300. https://doi.org/10.4049/jimmunol.1601279

12. Graef T, Moesta AK, Norman PJ, Abi-Rached L, Vago L, Older Aguilar AM, et al. KIR2DS4 is a product of gene conversion with KIR3DL2 that introduced specificity for HLA-A*11 while diminishing avidity for HLA-C. Journal of Experimental Medicine. 2009;206:2557–72. https://doi.org/10.1084/jem.20091010

13. Hsu KC, Liu X-R, Selvakumar A, Mickelson E, O'Reilly RJ, Dupont B. Killer Ig-Like Receptor Haplotype Analysis by Gene Content: Evidence for Genomic Diversity with a Minimum of Six

Basic Framework Haplotypes, Each with Multiple Subsets. The Journal of Immunology. 2002;169:5118–29. https://doi.org/10.4049/jimmunol.169.9.5118

14. Jiang W, Johnson C, Jayaraman J, Simecek N, Noble J, Moffatt MF, et al. Copy number variation leads to considerable diversity for B but not A haplotypes of the human KIR genes encoding NK cell receptors. Genome Res. 2012;22:1845–54. https://doi.org/10.1101/gr.137976.112
