# Supplementary material for: Examining the association between fetal HLA-C, maternal KIR haplotypes and birth weight
Source: PLoS Genet. 2026 Apr 20;22(4):e1012102. doi: 10.1371/journal.pgen.1012102 (PMC13095029; doi:10.1371/journal.pgen.1012102)
Supplement: Attachment — Submitted filename: ResponseToReviewers_PLOSGen_2026_03_20.pdf [file pgen.1012102.s013.pdf]

Responses to Editor and Reviewers

Submission ID: PGENETICS-D-25-00434R1
Corresponding author: Caitlin S. Decina
Title: Examining the association between fetal HLA-C, maternal KIR haplotypes and birth weight

We are grateful for the consideration of our revised manuscript and evaluation of our responses following initial peer review. We are pleased to see that Reviewer #2 feels that their comments have now been appropriately addressed but regretful that Reviewer #1 has remaining concerns about our manuscript, particularly with regard to the accuracy of our imputed KIR types, in addition to the lack of data on gestational age and leaving out the extremes of the normal birth weight distribution. Our response to each of these points is below, with any text added to the manuscript stated beneath its corresponding response; page and line numbers refer to the clean resubmitted manuscript document. New text introduced to the manuscript has been added to a new marked-up version showing tracked changes (revisions from initial peer review incorporated and only new additions highlighted); this has been uploaded as a related file.

1. The use of KIR*IMP and evaluating imputation accuracy.

We appreciate that data quality has great importance and vigilance in this regard is understandable. We assert, however, that although KIR imputation may not be the gold standard for the determination of genotypes, KIR*IMP is a standard and accepted method for imputing KIR loci. The KIR*IMP method and software has been previously validated in an independent dataset, and was peer reviewed and published in a top tier genetics journal (AJHG) which has been cited more than 50 times (Vukcevic et al. 2015). The reviewer's suggestion to directly type 25% of our sample to verify its accuracy is akin to a request to revalidate the method – an exercise that would be both logistically infeasible and unnecessary as KIR*IMP has been used to impute KIR loci successfully in top tier publications including Mack et al. 2020 Science Translational Medicine; Scepanovic et al 2019 Microbiome; Bian et al 2022 Brain Communications; Gilchrist et al 2022 Nature Communications; and many others, without the requirement to revalidate the method. Lastly, we highlight that KIR*IMP is readily, and freely, available from https://imp.science.unimelb.edu.au/kir/ including documentation/instructions for use.

Following the suggestion of the editors, we instead provide a quantitative demonstration of the robustness of our findings by performing simulations (with accompanying R code) examining the effect of imputation accuracy on power to detect a main effect of maternal *KIR2DS1* as well as an interaction between maternal *KIR2DS1* and fetal *HLA-C2* on offspring birth weight. In short, because our study is an order of magnitude larger than the original Hiby et al. 2014 study, the simulations indicate that we maintain very high power to detect main effects and interactions even with substantially reduced KIR imputation accuracy (simulated as low as 70%). Note that the actual accuracy of imputation in our study is likely to be far greater than this (~95% as shown in **Table 4** of the manuscript). We describe the full simulation procedure below.

Estimating the proportion of variance explained by the main effect and interaction reported in Hiby et al. (2014)

Hiby et al. report that the frequency of *KIR2DS1* in the English population is approximately p = 0.2. It follows that approximately 0.2^2 + 2*0.2*0.8 = 0.36 of mothers should be positive for *KIR2DS1*. Hiby et al. report that presence of *KIR2DS1* is associated with an 84g increase in birth weight. Thus, the locus explains approximately $2*p*(1-p)*\beta^2$ = 0.36*(1 - 0.36)*84^2 = 1625.7024 g^2 variance. Assuming the standard deviation of birth weight is roughly 500g, the phenotypic variance is around 250000g^2. This translates to the locus explaining 0.65% of the variance. Most complex trait loci explain <0.1% of the variance in quantitative traits and this has certainly been the case for birth weight, however, a locus that explains 0.65% of the variance is not outside the realms of possibility.

In order to estimate the proportion of variance explained by the interaction reported in Hiby et al., we need to work out the expected proportion of pregnancies where the offspring possesses more *HLA-C2* alleles than their mother. Assume that the frequency of *HLA-C2* is p = 0.3. This is equivalent to saying that 30% of offspring carry an *HLA-C2* allele from their father. For the 30% of offspring who carry this allele transmitted from their father, their mothers can be homozygous for the other allele with probability (1 - p)^2 = 0.7^2 = 0.49, or heterozygous and transmit the other allele to their offspring with probability p*(1 – p) = 0.7*0.3 = 0.21. Thus, the expected proportion of pregnancies where the offspring possesses more *HLA-C2* alleles than their mother is 0.3*(0.49 + 0.21) = 0.21.

As explained above, we expect 36% of mothers to be positive for *KIR2DS1*. Assuming independence, the proportion of offspring who should carry an *HLA-C2* allele from their father and have a mother positive for *KIR2DS1* = 0.21*0.36 = 0.0756. Hiby et al. report that the interaction confers a 245g increase in birth weight. Thus, the locus explains 0.0756*(1 – 0.0756)*245^2 = 4194.8 g^2 variance. This translates to the interaction explaining 1.68% of the phenotypic variance.

Estimating Power to Detect a Main Effect of KIR2DS1 and an Interaction Between Maternal KIR2DS1 and Fetal HLA-C2 In Our Study Via Simulation

We simulated genotypes at the *KIR2DS1* locus assuming an allele frequency of p = 0.2 for 10,602 mothers. We simulated a phenotype (offspring birth weight) where maternal *KIR2DS1* explained 0.65% of the phenotypic variance in offspring birth weight according to a dominant model. We simulated so that birth weight had unit variance. We then ran a test of association regressing offspring birth weight on the number of copies of the risk allele in mothers. We calculated power across 1000 simulations.

We examined the effect of imputation error on the power to detect an association. We parameterized accuracy as the probability of correctly imputing the correct *KIR2DS1* genotype. Genotypes that “failed” imputation were randomly changed to one of the other two genotype categories. We estimated power (two tailed α = 0.05) across 1000 simulations where birth weight was regressed on the number of copies of the *KIR2DS1* allele. **Table A** shows that we would expect to maintain high power to detect an effect of *KIR2DS1* even with substantial decreases in imputation accuracy (i.e. far larger than what would be the case in our study). Intuitively, this is because our study is an order of magnitude larger than the original Hiby et al. publication.

Table A. Estimated power to detect an association between maternal *KIR2DS1* and offspring birth weight with varying levels of imputation accuracy using simulation.

KIR Imputation Accuracy	Power To Detect Association
100%	100%

95%	100%
90%	100%
85%	99.9%
80%	99.5%
75%	95.9%
70%	89.5%

To simulate the interaction, we assumed that the frequency of the birth weight increasing biallelic *HLA-C2* allele was p = 0.3. We simulated transmission of the variant in 10,602 parent-offspring trios and independently simulated the biallelic *KIR2DS1* locus as before in mothers. We simulated offspring birth weight to have unit variance and assumed that the interaction between maternal *KIR2DS1* and offspring *HLA-C2* explained 1.68% of the phenotypic variance. Specifically, offspring birth weight increased only when mothers were positive for *KIR2DS1* and their offspring carried more *HLA-C2* alleles than their mothers. For each replicate, we regressed offspring birth weight on three terms, the number of copies of *KIR2DS1* in mothers, a binary term indicating whether the offspring carried more *HLA-C2* alleles than their mothers, and a term representing an interaction between the two terms. We calculated the significance of the interaction term. Power was defined as the number of statistical tests of the interaction term reaching significance across 1000 simulations (two tailed $\alpha = 0.05$). We examined the effect of imputation error on the power to detect association as before.

Again, power to detect the interaction was extremely high and did not decrease measurably even with the addition of substantial imputation error in maternal *KIR2DS1* (power > .99 with only 80% accuracy).

R code detailing the simulations is appended at the end of this document.

We have added this material as supplementary information to the manuscript under the Supporting Information section, titled “S2 Supplementary Information”. We have also acknowledged this briefly in the manuscript as an additional sensitivity analysis within the limitations of our discussion:

Discussion, page 23, lines 485-492: “Adding further support for our study findings relative to our imputation quality, we performed a sensitivity analysis using simulations to examine the effect of varying imputation accuracy on the power to detect a main effect of maternal *KIR2DS1* as well as an interaction between maternal *KIR2DS1* and fetal *HLA-C2* on offspring birth weight. The results indicated that we maintain very high power to detect main effects and interactions in our study, even with substantially reduced KIR imputation accuracy (simulated as low as 70%; see **S2 Supplementary Information**). This can be attributed to having a sample size an order of magnitude larger than the original Hiby et al. study.”

2. Lack of data on gestational age.

Regarding the lingering concern about the lack of gestational age data in the populations used in our study, this is only the case for the UK Biobank cohort due to the self-reported nature of the birth weight data – a cohort that represents less than one third of the total sample used for association testing. Gestational age is simply not available in this cohort but as we reported in our initial response to reviewer comments (comment #1 in the original response document), our reasonable attempt to investigate the impact of these missing data was done, as requested by Reviewer #1, with a sensitivity analysis leaving this cohort out of our meta-analysis. The overall associations remained null with large confidence intervals, ultimately showing no difference to the conclusions made in the manuscript when UK Biobank is included in the meta-analysis. This information was added to the manuscript as a

supplement for comprehensiveness and transparency. We maintain that the absence of gestational age data is not causing undue influence on the results, that removal of the UK Biobank cohort from the reported results is thus not necessary, and that the conclusions of the manuscript stand despite the lack of uniformity for this variable in the populations used for analysis.

3. Leaving out the extremes of the normal birth weight distribution.

As stated in our responses to the initial reviewer comments with respect to the birth weight extremes (comment #3 in the original response document), even within the Hiby et al. 2014 study, not all birth weights were included in their analysis. Their study included a sample of all pregnancies >5th centile, excluding pregnancies with fetal growth restriction and pre-eclampsia. Their study thus included high birth weights (of an undisclosed upper limit), though of the spectrum of birth weights included, most fell within the normal range (994/1,316, or 76%). While not an exact 1:1 replication with respect to the range of birth weights included, our study tests their same primary asserted relationship of a birth weight-raising effect of the presence of maternal *KIR2DS1* in combination with fetal *HLA-C2* of paternal origin when tested under the assumptions of a linear regression model using birth weight as a continuous variable (in grams). We reiterate that given their finding of a large increase in birth weight of ~250g for the combination of maternal *KIR2DS1* and fetal *C2* of paternal origin, we would expect to see some effect of increased birth weight even within the spectrum of normal birth weight (as we have used) if this relationship were true. In the sensitivity analyses we performed as part our initial response to comments testing the inclusion of previously excluded birth weight outliers in our UKB sample, where birth weight had been limited to a specific range of 2.5kg – 4.5kg, we saw no clearer association in the direction of increased birth weight among the tested KIR and HLA combinations.

We have now addressed the reviewer's foremost concern – that of the impact of KIR imputation accuracy. The results of our simulations indicate strong power to detect the previous association from Hiby et al. 2014 if it exists, even in cases of suboptimal accuracy. This provides confidence that the quality of the genotypes our findings rest upon and the power of the study overall is reliable. Given the results of our simulations and the sensitivity analyses we have performed (reiterated above), it is unreasonable to expect that variability in gestational age or the spectrum of birth weights with respect to the original study are material differences as to why we were unable to reproduce the original association.

We trust that our responses to the remaining comments above, which include further analyses, support the robustness of our work. We look forward to the journal's final decision on our manuscript.

R Code

```
###MAIN EFFECT###
set.seed(123)

### PARAMETERS
n <- 10602
p <- 0.2
nsim <- 1000
alpha <- 0.05
var_explained <- 0.0065

### Genotype probabilities under HWE
geno_probs <- c((1-p)^2, 2*p*(1-p), p^2)

### Dominant indicator variance
q <- 1 - (1-p)^2
varD <- q * (1 - q)

### Effect size giving 0.65% variance explained
beta <- sqrt(var_explained / varD)

### Residual SD so phenotype variance = 1
sigma <- sqrt(1 - var_explained)


##################################################
# FUNCTION: run simulation with optional error
##################################################

simulate_power <- function(accuracy = 1){

  pvals <- numeric(nsim)

  for(i in 1:nsim){

    ### Simulate genotype (0,1,2)
    geno <- sample(0:2, n, replace=TRUE, prob=geno_probs)

    ### Dominant model for true phenotype
    D <- ifelse(geno > 0, 1, 0)

    ### Simulate phenotype
    y <- beta * D + rnorm(n,0,sigma)

    ### Introduce genotyping error
    geno_obs <- geno

    wrong <- runif(n) > accuracy

    if(any(wrong)){
      geno_obs[wrong] <- sapply(geno_obs[wrong], function(g){
        sample(setdiff(0:2, g), 1)
      })
```

```
    }

    ### Association test (additive model)
    fit <- lm(y ~ geno_obs)

    pvals[i] <- summary(fit)$coef[2,4]
  }

  power <- mean(pvals < alpha)

  return(power)
}


#################################################
# POWER WITHOUT GENOTYPING ERROR
#################################################

power_true <- simulate_power(accuracy = 1)

power_true


#################################################
# POWER WITH DIFFERENT GENOTYPING ACCURACY
#################################################

accuracy_values <- seq(0.7,1,0.05)

power_results <- sapply(accuracy_values, simulate_power)

results <- data.frame(
  accuracy = accuracy_values,
  power = power_results
)

results
```

###R CODE FOR INTERACTION###

```
set.seed(123)

#############################
# PARAMETERS
#############################

n <- 10602
p_kir <- 0.2
p_hla <- 0.3
var_explained <- 0.0168
nsim <- 1000
alpha <- 0.05
```

```
##############################
# GENOTYPE SIMULATION
##############################

simulate_genotype <- function(n, p){
  probs <- c((1-p)^2, 2*p*(1-p), p^2)
  sample(0:2, n, replace=TRUE, prob=probs)
}


##############################
# HLA TRANSMISSION
##############################

simulate_trios <- function(n, p){

  mother <- simulate_genotype(n, p)
  father <- simulate_genotype(n, p)

  transmit <- function(g){
    sapply(g, function(x){
      if(x==0) return(0)
      if(x==2) return(1)
      rbinom(1,1,0.5)
    })
  }

  off_m <- transmit(mother)
  off_f <- transmit(father)

  offspring <- off_m + off_f

  list(mother = mother,
       offspring = offspring)
}


##############################
# PHENOTYPE SIMULATION
##############################

simulate_phenotype <- function(kir_mother, hla_mother, hla_offspring){

  kir_dom <- as.numeric(kir_mother > 0)

  hla_diff <- as.numeric(hla_offspring > hla_mother)

  interaction <- kir_dom * hla_diff

  vg <- var(interaction)

  beta <- sqrt(var_explained / vg)

  genetic <- beta * interaction
```

```
  residual_sd <- sqrt(1 - var_explained)

  y <- genetic + rnorm(length(genetic), 0, residual_sd)

  return(list(y=y,
              kir_dom=kir_dom,
              hla_diff=hla_diff))
}


##############################
# GENOTYPING ERROR FUNCTION
##############################

introduce_error <- function(g, accuracy){

  g_obs <- g

  incorrect <- runif(length(g)) > accuracy

  if(any(incorrect)){
    g_obs[incorrect] <- sapply(g_obs[incorrect], function(x){
      sample(setdiff(c(0,1,2), x), 1)
    })
  }

  g_obs
}


##############################
# ASSOCIATION TEST
##############################

test_interaction <- function(y, kir_add, hla_diff){

  fit <- lm(y ~ kir_add + hla_diff + kir_add:hla_diff)

  summary(fit)$coefficients["kir_add:hla_diff",4]
}


##############################
# POWER WITHOUT ERROR
##############################

pvals <- numeric(nsim)

for(i in 1:nsim){

  kir_mother <- simulate_genotype(n, p_kir)

  trio <- simulate_trios(n, p_hla)
```

```
  hla_mother <- trio$mother
  hla_offspring <- trio$offspring

  sim <- simulate_phenotype(kir_mother, hla_mother, hla_offspring)

  pvals[i] <- test_interaction(sim$y,
                               kir_mother,
                               sim$hla_diff)
}

power_no_error <- mean(pvals < alpha)



##############################
# POWER WITH GENOTYPE ERROR
##############################

accuracy <- 0.80

pvals_error <- numeric(nsim)

for(i in 1:nsim){

  kir_true <- simulate_genotype(n, p_kir)

  trio <- simulate_trios(n, p_hla)

  hla_mother <- trio$mother
  hla_offspring <- trio$offspring

  sim <- simulate_phenotype(kir_true, hla_mother, hla_offspring)

  kir_obs <- introduce_error(kir_true, accuracy)

  pvals_error[i] <- test_interaction(sim$y,
                                     kir_obs,
                                     sim$hla_diff)
}

power_with_error <- mean(pvals_error < alpha)


##############################
# RESULTS
##############################

cat("Power without genotyping error:", power_no_error, "\n")
cat("Power with KIR2DS1 genotyping accuracy =", accuracy, ":",
power_with_error, "\n")
```
